# Glibenclamide Alleviates LPS-Induced Acute Lung Injury through NLRP3 Inflammasome Signaling Pathway

**DOI:** 10.1155/2022/8457010

**Published:** 2022-02-11

**Authors:** Jie Yang, Jiawen Yang, Xiaofang Huang, Huiqing Xiu, Songjie Bai, Jiahui Li, Zhijian Cai, Zhanghui Chen, Shufang Zhang, Gensheng Zhang

**Affiliations:** ^1^Department of Critical Care Medicine, Second Affiliated Hospital, Zhejiang University School of Medicine, Hangzhou 310009, China; ^2^Institute of Immunology, and Department of Orthopaedics of the Second Affiliated Hospital, Zhejiang University School of Medicine, Hangzhou 310009, China; ^3^Department of Critical Care Medicine, Qilu Hospital, Cheeloo College of Medicine, Shandong University, Jinan 250012, China; ^4^Zhanjiang Institute of Clinical Medicine, Zhanjiang Central Hospital, Guangdong Medical University, Zhanjiang 524045, China; ^5^Department of Cardiology, Second Affiliated Hospital, Zhejiang University School of Medicine, Hangzhou 310009, China; ^6^Key Laboratory of the Diagnosis and Treatment of Severe Trauma and Burn of Zhejiang Province, China; ^7^Zhejiang Province Clinical Research Center for Emergency and Critical Care Medicine, China

## Abstract

Glibenclamide displays an anti-inflammatory response in various pulmonary diseases, but its exact role in lipopolysaccharide- (LPS-) induced acute lung injury (ALI) or acute respiratory distress syndrome (ARDS) remains unknown. Herein, we aimed to explore the effect of glibenclamide in vivo and in vitro on the development of LPS-induced ALI in a mouse model. LPS stimulation resulted in increases in lung injury score, wet/dry ratio, and capillary permeability in lungs, as well as in total protein concentration, inflammatory cells, and inflammatory cytokines including IL-1*β*, IL-18 in bronchoalveolar lavage fluid (BALF), and lung tissues, whereas glibenclamide treatment reduced these changes. Meanwhile, the increased proteins of NLRP3 and Caspase-1/p20 after LPS instillation in lungs were downregulated by glibenclamide. Similarly, in vitro experiments also found that glibenclamide administration inhibited the LPS-induced upregulations in cytokine secretions of IL-1*β* and IL-18, as well as in the expression of components in NLRP3 inflammasome in mouse peritoneal macrophages. Of note, glibenclamide had no effect on the secretion of TNF-*α* in vivo nor in vitro, implicating that its anti-inflammatory effect is relatively specific to NLRP3 inflammasome. In conclusion, glibenclamide alleviates the development of LPS-induced ALI in a mouse model via inhibiting the NLRP3/Caspase-1/IL-1*β* signaling pathway, which might provide a new strategy for the treatment of LPS-induced ALI.

## 1. Introduction

Acute lung injury (ALI) or acute respiratory distress syndrome (ARDS), a common disease in intensive care unit (ICU), is the consequence of biased inflammatory response to various causes including sepsis, trauma, and ventilation [1–3]. Although supportive treatment and intensive care are developing, there have been no clinically effective pharmacologic therapies and the prognosis of ALI/ARDS remains poor with a high morbidity and mortality [1, 3]. Thus, to further explore the underlying mechanisms and the potential treatment approaches for ALI/ARDS is necessary and urgent.

NLRP3, belonging to a family of NLRs, is one of the immediate responses of the innate immune system. It is postulated that a two-step mechanism is required for the full activation of the NLRP3 inflammasome [4]. The first is a priming step that is initiated by pathogen-associated molecular patterns (PAMPs) or damage-associated molecular patterns (DAMPs), resulting in the upregulations of pro-IL-1*β*, pro-IL-18, and the components of the inflammasome. The second is an activation step which is the assembly of these components into the inflammasome structure and then to produce mature proinflammatory interleukins. Recent research has described the function of the NLRP3 inflammasome in various pulmonary diseases including respiratory infections, chronic obstructive pulmonary disease, and asthma [5, 6]. Currently, the role of NLRP3 inflammasome in the development of multiple types of ALI is also reported, and the main pathogenic mechanisms include the following: (1) increased permeability of alveolar epithelial and barrier dysfunction; (2) overproduction of cytokines including IL-6, IL-1*β*, and TNF-*α*; and (3) involvement of tissue remodeling and pulmonary fibrosis in the late stage of ALI [7–9]. These results imply that the NLRP3 inflammasome participates in the pathogenesis of ALI/ARDS.

Glibenclamide, besides as a kind of hypoglycemic drug, displays an anti-inflammatory role in many diseases from respiratory, urinary, heart, and central nervous systems [10]. Given the fact of ALI/ARDS considered as an inflammatory disorder and the anti-inflammatory activity of glibenclamide, hence, glibenclamide might be against the development of ALI/ARDS in theory. In fact, a large number of studies have indicated that glibenclamide involves in the regulation of inflammation in different animal models of ALI, including oleic acid-, ozone-, radiation-, hemorrhagic shock-, and ventilator-induced ALI [11–14]. However, no such studies are reported concerning the role of glibenclamide in LPS-induced ALI. LPS, a major constituent of the outer membrane of Gram-negative bacteria, acts as one of the common causes in the pathogenesis of sepsis, septic shock, and sepsis-related ALI/ARDS [15]. In addition, animal model of LPS-induced ALI is widely used as a clinically relevant model of Gram-negative bacteria-related ALI/ARDS [16–18]. Therefore, it would be of great clinical significance to explore whether glibenclamide can ameliorate LPS-induced ALI.

Taken together, we proposed the hypothesis that glibenclamide has a protective effect on LPS-induced ALI/ARDS, which might be associated with its inhibition of NLRP3 inflammasome signaling pathway. Thus, we herein attempted to confirm this hypothesis via in vivo and in vitro experiments in a mouse model of LPS-induced ALI and for the first time found that glibenclamide alleviated the development of LPS-induced ALI in a mouse model via inhibiting the NLRP3/Caspase-1/IL-1*β* signaling pathway, which might provide a new strategy for the treatment of LPS-induced ALI.

## 2. Materials and Methods

### 2.1. Animals and Study Design

Male C57BL/6 mice (6-8 weeks) were purchased from the Shanghai SLAC Laboratory Animal Co., Ltd. (Shanghai, China). All experimental procedures were conducted in accordance with the ethics committee of the animal laboratory of Zhejiang University. Mice were divided into four groups (*n* = 6/group): control (Con) group, glibenclamide (Gly) group, LPS group, and LPS+glibenclamide (LPS+Gly) group. Glibenclamide (Sigma-Aldrich, St. Louis, MO, USA) was diluted in DMSO for 100 mg/ml concentration according to the instruction manual. Glibenclamide was given intraperitoneally for 3 days before LPS administration, whereas DMSO was used as vehicle. LPS (Sigma-Aldrich, St. Louis, MO, USA) was injected into the trachea of mice with a microsyringe to establish ALI model, while PBS was used as vehicle. After intratracheal instillation, mice were kept vertical for at least 1 min to ensure the distribution of the PBS or LPS in the lungs. Twenty-four hours later after LPS administration, the mice were sacrificed for experiments. Bronchoalveolar lavage fluid (BALF) was collected with PBS via a tracheal catheter as described in our previous study [19]. After centrifugation, the supernatant and cells were separated for further experiments. The lung tissues were collected for further analysis.

### 2.2. Lung Histology and Immunohistochemistry Analysis

The lung tissues fixed in 4% paraformaldehyde were embedded in paraffin and then sliced at a thickness of 4 *μ*m for hematoxylin and eosin (H&E) staining. The histology scoring system was used to evaluate lung injury [11]. Four pathological parameters were scored as previously described: (1) alveolar congestion, (2) hemorrhage, (3) leukocyte infiltration, and (4) thickness of alveolar wall/hyaline membrane formation. Each category was graded using a 4-pointscale: 0: minimal damage, 1: mild damage, 2: moderate damage, and 3: maximal damage. The total histology score was expressed as the sum of the score for all parameters. Three slides of each mouse were prepared for evaluation.

Immunohistochemistry (IHC) was performed to determine the protein expression of NLRP3. The paraffin sections were pretreated at 62°C for 30 min, then dewaxed in xylene, hydrated, and washed. Hydrogen peroxide solution was used to inhibit the endogenous peroxidase. The sections were incubated overnight at 4°C with anti-NLRP3 antibody (Abclonal, Wuhan, China) (1 : 100). Then, membranes were washed thoroughly with phosphate-buffered saline solution. The secondary antibodies (Tuling, Hangzhou, China) were added and incubated at 37°C for 30 min. Diaminobenzidine was added, and the sections were counterstained with hematoxylin to visualize the reaction products. All the sections were semiquantitatively analyzed by the ImageJ software. The integrated IOD/area (density mean) was measured by evaluating the staining in images at ×200 magnification. Three slides of each mouse were prepared for evaluation.

### 2.3. Real-Time Quantitative Polymerase Chain Reaction (RT-qPCR)

Total RNA was extracted from lung tissues or cells using Trizol (Thermo Fisher Scientific). cDNA was synthesized using a cDNA synthesis kit (Takara, Dalian, Liaoning, China) following the manufacturer's instructions. For mRNA detection, *β*-actin was used as the reference housekeeping gene. Real-time PCR was conducted using SYBR Green (TaKaRa) with an Applied Biosystems 7500 real-time PCR system (Thermo Fisher Scientific). The primer sequences used are shown as follows: mouse NLRP3 sense primer: 5′-TCACAACTCGCCCAAGGAGGAA-3′ and mouse NLRP3 antisense primer: 5′-AAGAGACCACGGCAGAAGCTAG-3′ and mouse *β*-actin sense primer: 5′-GGCTGTATTCCCCTCCATCG-3′ and mouse *β*-actin antisense primer: 5′-CCAGTTGGTAACAATGCCATGT-3′.

### 2.4. Western Blot Analysis

Cell or tissue lysate was resuspended in 5× SDS loading buffer, subsequently incubated at 100°C for 5 min and centrifuged at 12,000 × g for 10 min. Protein concentrations were detected using a BCA Protein Assay Kit (Thermo Fisher Scientific). A total of 20 *μ*g of protein from the tissue or cell lysate was separated by SDS-PAGE gel (Thermo Fisher Scientific) and then transferred onto polyvinylidene difluoride (PVDF) membranes (Millipore). The membrane was blocked using 5% nonfat milk for 2 h at room temperature and then incubated with appropriate primary antibodies: anti-NLRP3 (Abclonal, Wuhan, China) (1 : 1,000) and anti-Caspase-1 (Abclonal, Wuhan, China) (1 : 1,000) in blocking buffer overnight at 4°C. Anti-*β*-actin (HuaBio, Shanghai, China) (1 : 2,000) was used as a loading control. After washing three times with PBST, the membranes were incubated with HRP-conjugated secondary antibodies for 1.5 h at room temperature. The bands were detected using an ECL kit (MultiSciences, Hangzhou, Zhejiang, China).

### 2.5. ELISA Assays

The levels of IL-1*β*, IL-18, and TNF-*α* concentrations in lung tissues, BALF, and cell supernatant were analyzed using ELISA Kit (BioLegend, San Diego, CA, USA), according to the manufacturer's protocol.

### 2.6. Mouse Lung Wet/Dry Ratio Assay

Twenty-four hours after intratracheal instillation of LPS, mice were killed and the lobes of the right lungs were excised after removal of excess blood and then weighed to obtain the “wet” weight. Subsequently, the lungs were dried in an oven at 60°C for 72 h for “dry” weight.

### 2.7. The Measurement of BALF

Total cell number of BALF was counted, and total protein concentration in BALF was determined using a BCA assay Kit (Thermo Fisher Scientific) according to the manufacturer's instructions. The inflammatory cells in BALF were analyzed with a Cytoflex machine (Beckman Coulter), and the following fluorescence-conjugated antibodies were used for the experiment: PE-conjugated anti-mouse CD11b (BioLegend, San Diego, CA, USA), FITC-conjugated anti-mouse F4/80 (BioLegend, San Diego, CA, USA), and BV650-conjugated anti-mouse LY-6G (Invitrogen, Carlsbad, CA, USA).

### 2.8. Mouse Alveolar-Capillary Leakage Assay

Twenty-four hours after LPS administration, the mice were injected with 20 mg/kg Evans blue solution by the tail vein. Two hours later, the mice were exsanguinated through the heart with syringe. Then, the lungs were removed and placed in 100 mg/ml formamide (Sigma-Aldrich, St. Louis, MO, USA). The tissues were incubated at 60°C for 24 h, and the absorbance of formamide was measured at 620 nm.

### 2.9. Isolation and Purification of Mouse Peritoneal Macrophages

C57BL/6 mice at 8-week-old were i.p. injected with 2 ml of 3% sterile thioglycolate medium (BD Biosciences, Sparks, MD), and peritoneal macrophages (PMs) were extracted three days later. To isolate and purify the PMs, each mouse was euthanized with 40 mg/kg pentobarbital sodium and soaked in 75% ethanol for 3 min. The outer layer of the peritoneum was incised with scissors; 15 ml RPMI 1640 was injected intraperitoneally into mice with a 20 ml syringe. The intraperitoneal fluid was collected into the tube with a 20 ml syringe after gently massaging the peritoneum and centrifuged at 4°C for 250 × g for 5 min. The supernatant was discarded, and the sediment was suspended in RPMI 1640 medium supplemented with 10% fetal bovine serum and 1% penicillin/streptomycin. The cells were then added to 12-well cell culture plates as needed to obtain a density of 5 × 10^6^ cells/well and cultured for 2 h at 37°C in 5% CO2. Then, nonadherent cells were removed by gentle washing with PBS three times. The isolated macrophages were prepared for experiments in vitro.

### 2.10. Cell Proliferation Assay

Cell Counting Kit-8 kits (CCK-8, TransGen, Beijing, China) were used to evaluate PM proliferation. PMs were plated into 96-well cell culture plates at a density of 1 × 10^4^ cells/well for 24 h at 37°C and then treated with different concentrations of glibenclamide (0~200 *μ*M) for 24 h. The viability was assayed at 24 h by using a Cell Counting Kit-8 assay (TransGen, Beijing, China).

### 2.11. Inflammasome Activation Assays

PMs were seeded at 5 × 10^6^/ml in 12 well-cell culture plates. The overnight medium was replaced on the following day, and cells were primed with 2 *μ*g/ml LPS for 6 h. Then, medium was added with glibenclamide (50 *μ*M) or DMSO (1 : 1,000) for another 6 h. Cells were finally stimulated with inflammasome activators: 2 mM adenosine triphosphate (Sigma-Aldrich, St. Louis, MO, USA) for 1 h. Supernatant was removed and analyzed using ELISA kits according to the manufacturer's instructions. Cells were collected for Western blot analysis.

### 2.12. Statistical Analysis

Statistical analysis was carried out using Graphpad Prism. The data were expressed as mean ± SD. The unpaired Student *t*-test was used for comparisons between two groups. Differences were considered significant at *P* < 0.05.

## 3. Results

### 3.1. Glibenclamide Attenuates LPS-Induced Lung Injury

**Figure 1:**
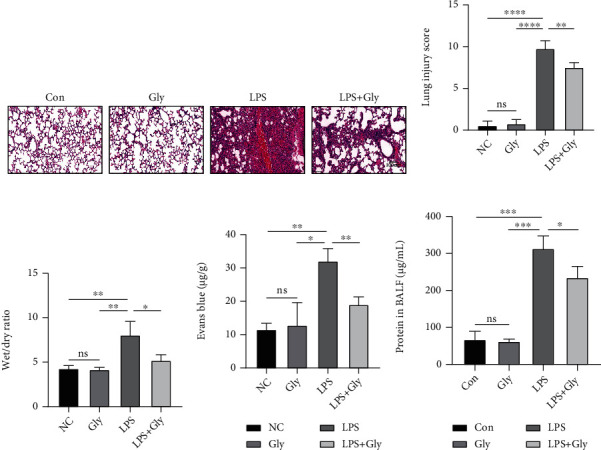
The effect of glibenclamide on the pathological injury in LPS-induced ALI. (a) The pathological alternations in lung tissues were evaluated with HE staining (*n* = 4). (b) Lung injury scores in four groups. (c) Wet/dry ratio in four groups (*n* = 4). (d) Alveolar-capillary leakage in four groups (*n* = 3). (e) The total protein concentration in BALF (*n* = 3). Scale bars, 100 *μ*m. Data are representative of three independent experiments (mean and SD). ns: not significant. ^∗^*P* < 0.05, ^∗∗^*P* < 0.01, and ^∗∗∗^*P* < 0.001 (unpaired Student's *t*-test).

After intratracheal instillation of LPS, mice showed greater diffuse alveolar damage, thickened alveolar wall, hemorrhage, and more inflammatory cell infiltration, whereas pretreatment with glibenclamide alleviated these pathological changes (Figure 1(a)). Correspondingly, glibenclamide treatment reduced the LPS-induced increases in inflammation score, the wet/dry ratio, alveolar-capillary leakage of lungs, and the concentration of total protein in BALF (Figures 1(b)–1(e)). These results suggested that glibenclamide attenuates the LPS-induced lung injury.

### 3.2. Glibenclamide Decreases LPS-Induced Lung Inflammation

**Figure 2:**
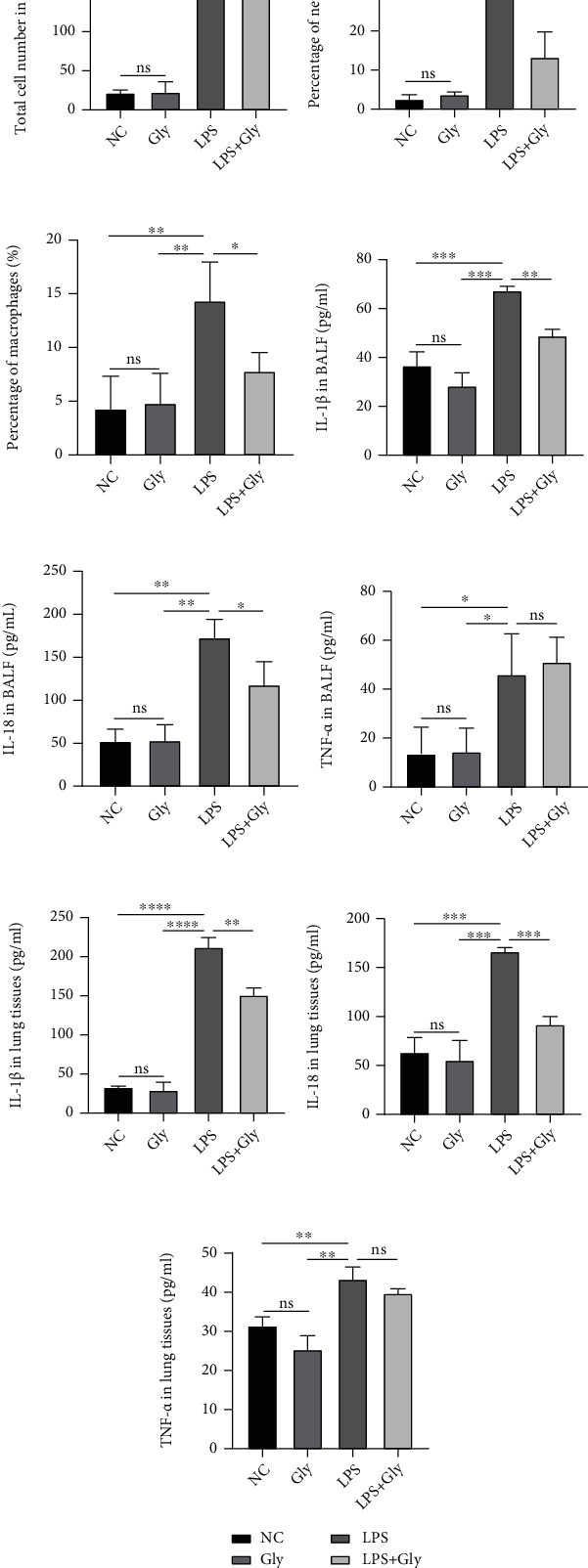
The effect of glibenclamide on the LPS-stimulated inflammatory response in lungs. (a) The total cell number counts in BALF (*n* = 3). (b, c) The percentage of neutrophils and macrophages is presented (*n* = 3). (d–i) The content of IL-1*β*, IL-18, and TNF-*α* in (d–f) BALF (*n* = 3) and (g–i) lung tissues (*n* = 3) was measured by ELISA. Data are representative of three independent experiments (mean and SD). ns: not significant. ^∗^*P* < 0.05, ^∗∗^*P* < 0.01, ^∗∗∗^*P* < 0.001, and ^∗∗∗∗^*P* < 0.0001 (unpaired Student's *t*-test).

In comparison with the LPS group, pretreatment with glibenclamide significantly reduced the total cell number and the percentage of neutrophils and macrophages in BALF (Figures 2(a)–2(c)). The levels of proinflammatory cytokines including IL-1*β* and IL-18 were also markedly downregulated in both BALF (Figures 2(d) and 2(e)) and lung tissues (Figures 2(g) and 2(h)). Surprisingly, glibenclamide treatment did not affect the LPS-mediated increase in the production of TNF-*α* either in BALF (Figure 2(f)) or in lung homogenates (Figure 2(i)).

### 3.3. Glibenclamide Suppresses the Expression of NLRP3 and Caspase-1 Activity

**Figure 3:**
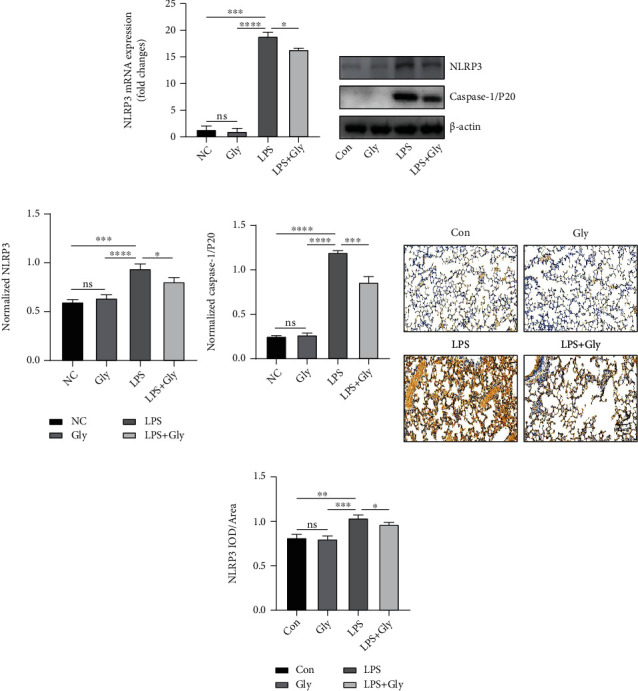
The inhibition role of glibenclamide in the NLRP3/Caspase-1/IL-1*β* signaling pathway induced by LPS. (a) Relative NLRP3 mRNA expression in lungs was measured by real-time PCR (*n* = 3). (b, c) Representative images of Western blot of NLRP3 and Caspase-1/p20 in the lungs and quantitative analysis (*n* = 3). (d, e) Representative images of immunohistochemical staining of NLRP3 in the lungs and quantitative analysis (*n* = 3). Scale bars, 100 *μ*m. Data are representative of three independent experiments (mean and SD). ns: not significant. ^∗^*P* < 0.05, ^∗∗^*P* < 0.01, ^∗∗∗^*P* < 0.001, and ^∗∗∗∗^*P* < 0.0001 (unpaired Student's *t*-test).

Based on the fact that glibenclamide can reduce the downstream products of NLRP3 signaling way, we supposed that glibenclamide could directly suppress the activation of NLRP3 inflammasome. Accordingly, we detected the expression of NLRP3, one of the main components of inflammasome, and Caspase-1/P20, a biologically active form of Caspase-1. Compared with the control group, the mRNA and protein levels of NLRP3 were obviously increased in the LPS group (Figures 3(a)–3(e)). Similarly, protein level of Caspase-1/P20 was also upregulated by LPS stimulation (Figures 3(b) and 3(c)). However, the elevated expressions of NLRP3 and Caspase-1/P20 in the LPS group were inhibited by glibenclamide treatment (Figures 3(a)–3(e)).

### 3.4. Glibenclamide Exerts Anti-Inflammatory Effect In Vitro

**Figure 4:**
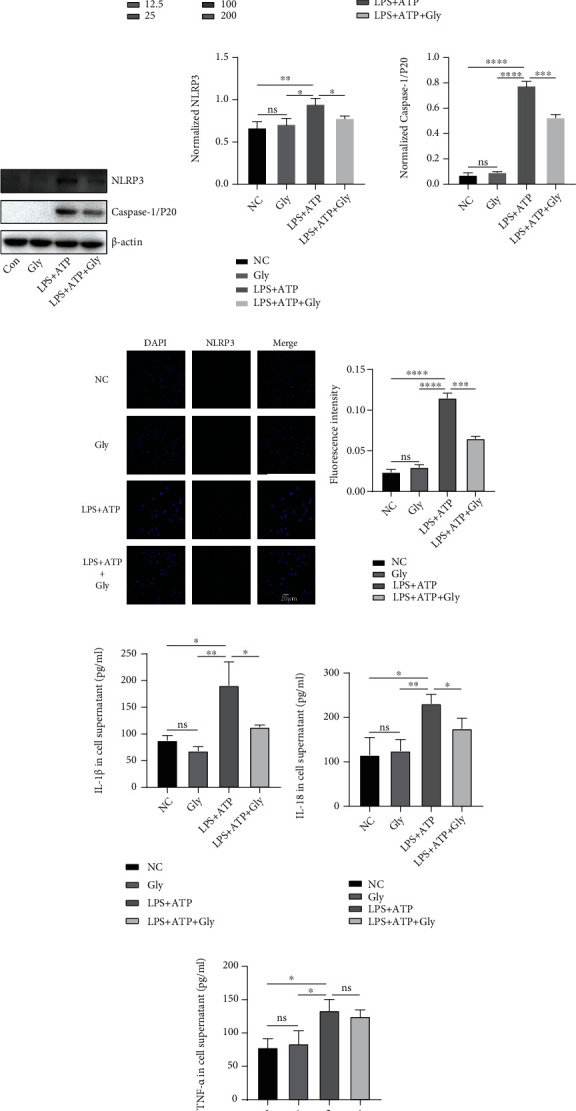
A protective role of glibenclamide in vitro inflammatory model. (a) CCK-8 assay was used to determine glibenclamide cytotoxicity (*n* = 3). (b) Relative NLRP3 mRNA expression in PMs was measured by real-time PCR (*n* = 3). (c, d) Representative images of Western blot of NLRP3 and Caspase-1/p20 in PMs and quantitative analysis (*n* = 3). (e) Representative immunofluorescence staining of NLRP3 in PMs. (f) Calculated percentage of NLRP3-positive nuclei (*n* = 3). (g–i) The level of IL-1*β*, IL-18, and TNF-*α* in cell supernatant was measured by ELISA (*n* = 3). Data are representative of three independent experiments (mean and SD). Scale bars, 20 *μ*m. ns: not significant. ^∗^*P* < 0.05, ^∗∗^*P* < 0.01, ^∗∗∗^*P* < 0.001, and ^∗∗∗∗^*P* < 0.0001 (unpaired Student's *t*-test).

Since the protective function of glibenclamide on LPS-induced ALI had been verified in vivo, then we further confirmed its effect with an in vitro model. Macrophages are key orchestrators of the inflammatory and repair responses in the lung [20]. In vitro inflammatory model established by LPS stimulation of macrophages including macrophage cell lines [21] or primary macrophages [22] can simulate the inflammatory process in vivo and is often used in the study of the mechanism of anti-inflammatory drugs [21, 22]. Herein, mouse peritoneal macrophages (PMs) were used as the in vitro cell model. First, we measured the in vitro cytotoxicity of glibenclamide to PMs. With the increased concentration, the cell viability decreased by approximately 36.9% at 100 *μ*M glibenclamide compared with the control (Figure 4(a)). Consequently, 50 *μ*M was recommended as the experimental dosage as glibenclamide at this concentration had no obvious cytotoxicity. PMs were first primed with LPS, then pretreated with glibenclamide, and lastly stimulated with the NLRP3 stimulus ATP. LPS stimulation promoted the expressions of NLRP3 mRNA and protein in PMs, and these upregulations were inhibited by glibenclamide treatment (Figures 4(b)–4(f)). Likewise, glibenclamide suppressed the activation of Caspase-1 (Figures 4(c) and 4(d)) and the release of IL-1*β* and IL-18 enhanced by LPS administration (Figures 4(g) and 4(h)). In contrast, the LPS-induced increase in expression of TNF-*α* was still not decreased after glibenclamide treatment (Figure 4(i)), which was consistent with the in vivo results (Figures 2(f) and 2(i)).

## 4. Discussion

In the current study, we revealed a previously unrecognized protective role of glibenclamide against LPS-induced acute lung injury. Glibenclamide could improve the pathological injury of lungs and attenuate pulmonary inflammation in a mouse model of LPS-induced ALI. Mechanistically, this protective effect is related to downregulations in the expression and activation of NLRP3/Caspase-1/IL-1*β* signaling pathway in vivo and in vitro. In addition, the inhibition in the inflammatory response by glibenclamide is partly specific to target NLRP3 inflammasome as it has no effect on the production/secretion of other inflammatory cytokines like TNF-*α*.

It is well-known that ALI/ARDS is characterized by sustained inflammation, excessive oxidative stress, and loss of alveolar-capillary membrane integrity, leading to increased lung microvascular permeability, alveolar edema, and leukocyte extravasations [1–3]. And so far, there have been considerable interventions described in the publications to prevent ALI/ARDS [23–25]. Among them, the anti-inflammatory property of glibenclamide in ALI/ARDS has been increasingly concerned and validated effectively in various animal models of ALI, including oleic acid-, ozone-, radiation-, hemorrhagic shock-, and ventilator-induced ALI [11–14]. In our study, administration of glibenclamide inhibits LPS-stimulated lung edema, vascular hyperpermeability damage, and inflammatory cell infiltration. These results indicate that in addition to the various models of ALI reported in previous studies [11–14], glibenclamide also exerts a protective role in the current model of LPS-induced ALI.

Based on our previous research, the mechanisms of glibenclamide underlying its anti-inflammatory role are summarized as follows [10]: (1) inhibiting the activation of NLRP3/IL-1*β* signaling, (2) downregulating the generation of reactive oxygen species, and (3) suppressing the migration of inflammatory cells like neutrophils and eosinophil. In the current research, the LPS-induced increases in levels of proinflammatory cytokines such as IL-1*β* and IL-18 and inflammatory cells like neutrophils and macrophages were decreased remarkably by glibenclamide in BALF and lung tissues. As IL-1*β* and IL-18 were known as the indicator of NLRP3 inflammasome induction [26–28], we hypothesized that the protective response of glibenclamide might be associated with its inhibition of NLRP3 inflammation. Indeed, a large number of studies have found that NLRP3 inflammasome plays an important role in ALI [14, 29]. Consistently, NLRP3/Caspase-1/IL-1*β* signaling was activated in vivo and in vitro after LPS or LPS plus ATP treatment, while the activation of NLRP3 inflammasome was inhibited by glibenclamide in our study. Thus, we confirmed that glibenclamide exerts its anti-inflammatory effect mainly by blocking NLRP3 signaling pathway.

Moreover, we noticed that the secretion of TNF-*α*, considered as an inflammasome-unrelated cytokine, was not impaired by glibenclamide. Consistent with previous studies [30, 31], our result found that glibenclamide did not affect LPS-stimulated TNF-*α* production, ruling out a more general anti-inflammatory effect by glibenclamide. These results suggest that the anti-inflammatory effect of glibenclamide is specifically related to NLRP3 inflammasome signaling pathway in LPS-induced ALI, though it could inhibit Th2 cytokines in ovalbumin-induced mouse model of asthma in our previous study [32].

Some limitations also exist in the current research. First, we did not determine the role of glibenclamide on other inflammasome complexes such as NLRP1, NLRC4, and AMI2. Second, we did not establish the model of LPS-induced ALI in NLRP3-/-mice, so the extent to which glibenclamide blocked the NLRP3 inflammasome has not been better clarified. Third, TNF-*α* is the product of activation of multiple inflammatory signaling pathways including the generic mitogen-activated protein kinases (MAPK) signaling pathway [33], the Janus kinase (JAK)/signal transducer and activator of transcription (STAT) pathway [34], and Hedgehog pathway [35]. Although the secretion of TNF-*α* was similar regardless of glibenclamide treatment, we did not observe whether glibenclamide has a role in the activation of these aforementioned multiple inflammatory signaling pathways. Thus, the evidence for the idea that the anti-inflammatory effect of glibenclamide is relatively specific to NLRP3 inflammasome seems not so solid. Last, we did not measure the blood glucose concentration in mice in the current study. Although the mouse model in the current study is different from the previous one which was ovalbumin-induced allergic asthma, we used the same therapeutic dose as before as 40 *μ*mol/kg for the treatment of LPS-induced ALI/ARDS and we did not make a fast pretreatment prior to glibenclamide administration as our previous study shown [32]. According to our previous work, we observed that the blood glucose concentration of mice was not affected at this concentration without a fast pretreatment prior to glibenclamide administration [32]; thus, we consider that the dose of glibenclamide at 40 *μ*mol/kg has no effect on blood glucose of mice in this study.

## 5. Conclusion

**Figure 5:**
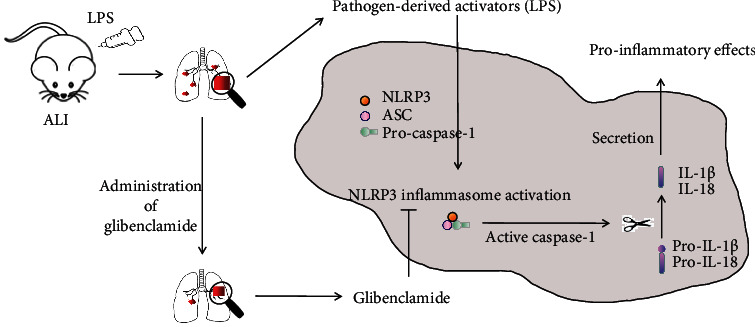
Schematic diagram of the role of glibenclamide on NLRP3 inflammasome in the pathogenesis of LPS-induced ALI. Glibenclamide blocked the activation of NLRP3 inflammasome induced by LPS and thus reduced the release of proinflammatory cytokines and then attenuated the lung injury consequently.

We demonstrated that glibenclamide alleviates LPS-induced ALI injury via an inhibition of inflammatory response, which is attributed to the suppression of NLRP3 inflammasome (Figure 5). Taken together, our results provide evidence that glibenclamide might be a promising candidate for the adjuvant therapy for LPS-induced ALI.

## Data Availability

All data of this study are available from the first author Yang G or the correspoinding author Zhang G if needed.
